# High day- and night-time temperatures affect grain growth dynamics in contrasting rice genotypes

**DOI:** 10.1093/jxb/erx344

**Published:** 2017-10-09

**Authors:** Wanju Shi, Xinyou Yin, Paul C Struik, Celymar Solis, Fangming Xie, Ralf C Schmidt, Min Huang, Yingbin Zou, Changrong Ye, S V Krishna Jagadish

**Affiliations:** 1 International Rice Research Institute (IRRI), DAPO Box, Metro Manila, Philippines; 2 Centre for Crop Systems Analysis, Department of Plant Sciences, Wageningen University & Research, AK Wageningen, The Netherlands; 3 Bayer Crop Science NV Innovation Center—Research, Technologiepark, Zwijnaarde (Gent), Belgium; 4 Southern Regional Collaborative Innovation Center for Grain and Oil Crops (CICGO), Hunan Agricultural University, Changsha, China; 5 Institute of Food Crops, Yunnan Academy of Agricultural Sciences, Yunnan, China; 6 Department of Agronomy, Kansan State University, Manhattan, KS, USA

**Keywords:** Chalkiness, grain filling, high day-time temperature, high night-time temperature, rice, starch metabolism enzymes, starch packaging

## Abstract

Rice grain yield and quality are predicted to be highly vulnerable to global warming. Five genotypes including heat-tolerant and susceptible checks, a heat-tolerant near-isogenic line and two hybrids were exposed to control (31 °C/23 °C, day/night), high night-time temperature (HNT; 31 °C/30 °C), high day-time temperature (HDT; 38 °C/23 °C) and high day- and night-time temperature (HNDT; 38 °C/30 °C) treatments for 20 consecutive days during the grain-filling stage. Grain-filling dynamics, starch metabolism enzymes, temporal starch accumulation patterns and the process of chalk formation were quantified. Compensation between the rate and duration of grain filling minimized the impact of HNT, but irreversible impacts on seed-set, grain filling and ultimately grain weight were recorded with HDT and HNDT. Scanning electron microscopy demonstrated irregular and smaller starch granule formation affecting amyloplast build-up with HDT and HNDT, while a quicker but normal amylopast build-up was recorded with HNT. Our findings revealed temporal variation in the starch metabolism enzymes in all three stress treatments. Changes in the enzymatic activity did not derail starch accumulation under HNT when assimilates were sufficiently available, while both sucrose supply and the conversion of sucrose into starch were affected by HDT and HNDT. The findings indicate differential mechanisms leading to high day and high night temperature stress-induced loss in yield and quality. Additional genetic improvement is needed to sustain rice productivity and quality under future climates.

## Teaser

High day and night temperatures affect the rate and duration of grain filling and starch packaging differently in a developing rice grain.

## Introduction

Global mean surface air temperature is predicted to increase by 1.0–3.7 °C by the end of the 21st century, which will potentially increase the frequency and magnitude of heat-stress events (IPCC, 2013). Under such climatic scenarios, rice plants are particularly vulnerable to heat stress during developmental periods of grain filling, leading to substantial reduction in yield and quality (Lobell and Gourdji, 2012; Lyman *et al.*, 2013). For example, in 2010 extremely high temperature after heading significantly reduced rice grain quality in many rice growing regions of Japan (Morita *et al.*, 2016). A heat wave with temperatures of 38 °C, well over the critical threshold of 33 °C (Bheemanahalli *et al.*, 2016), lasting for 10–20 d contributed to an estimated total paddy yield loss of 5.18 million tonnes in China (Yang and Li, 2005; Tian *et al.*, 2009).

Although an increase in global temperature has been well documented, a greater increase in night-time compared with day-time temperatures has been highlighted recently (Sillmann *et al.*, 2013). This differential increase in day and night temperature will result in a reduced diurnal temperature range, which has been shown to affect crop growth and development (Yin *et al.*, 1996; Peng *et al.*, 2004; Bahuguna and Jagadish, 2015). However, it is also reported that high day-time temperatures (HDT) in some of the major tropical rice growing regions are already close to the threshold beyond which yield begins to decline (Prasad *et al.*, 2006; Wassmann *et al.*, 2009). Additionally, the very first global mapping exercise differentiating vulnerability of rice growing regions to high day and night temperatures demonstrated regions that could be affected by HDT, high night-time temperature (HNT) or combined high night-time and day-time temperatures (HNDT) (Laborte *et al.*, 2012). By analysing yields obtained from 227 rice farms in six countries across South and Southeast Asia, Welch *et al.* (2010) pointed out that rice yields were differentially sensitive to increased maximum and minimum temperatures, supporting the above mapping exercise. Further, it has been shown that rice genotypes (both inbreds and hybrids) possess different response mechanisms to HNT compared with HDT in previous studies (Shi *et al.*, 2013; Jagadish *et al.*, 2015; Shi *et al.*, 2016; Bahuguna *et al.*, 2017). Hence, there is a need to study responses of rice plants exposed to HDT, HNT, and HNDT stresses in parallel, to determine the resilience of rice genotypes to these stresses for sustaining rice production across different geographical regions (Laborte *et al.*, 2012).

Exposure to increasing temperatures under HDT, HNT or combined HNDT under chamber (Yamakawa *et al.*, 2007; Cao *et al.*, 2016) or field (Shi *et al.*, 2013; Rehmani *et al.*, 2014; Bahuguna *et al.*, 2017) conditions during grain filling impairs grain growth, leading to poor seed-set and reduced single-grain weight. Changes in single-grain weight are often attributed to reduced carbohydrate supply (Dong *et al.*, 2014) and altered starch metabolism enzymes (Bahuguna *et al.*, 2017). Exposure to heat stress during grain filling also brings about poor grain quality, for example increased chalkiness of the grains (Ishimaru *et al.*, 2009; Lanning *et al.*, 2011). Chalk formation in rice grain is a result of loosely packed starch granules leading to air spaces between amyloplasts (Ashida *et al.*, 2009), which could result in a higher percentage of broken grains and significantly lower market value of the rice grain (Lyman *et al.*, 2013; Zhao and Fitzgerald, 2013). Chalky grains are usually classified into milky-white, basal-white, white-back and white-belly, based on the location of the chalk formation in the grain (Wada *et al.*, 2015). Determining the type and location of chalk formation is crucial, particularly under stress conditions (Lyman *et al.*, 2013). Despite their importance, comparative responses of rice to HDT and HNT independently and to combined HDT and HNT, affecting grain growth and starch packing over time and chalk formation, have not been systematically investigated. Hence a better understanding of the differential responses to HDT and HNT is needed to refine ongoing approaches towards developing heat stress-resistant rice cultivars.

Recent progress in improving heat tolerance in rice during flowering has resulted in fine mapping of an effective quantitative trait locus (QTL) on chromosome 4 (*qHTSF4.1*), which increased spikelet fertility by 15% at 38 °C compared with its susceptible parent, IR64 (Ye *et al.*, 2015). Both IR64 and its heat-tolerant near-isogenic line (HT NIL) introgressed with *qHTSF4.1* (Ye *et al.*, 2015) were tested to assess if the beneficial impact of heat tolerance observed during anthesis in the NIL could also reduce the impact of post-flowering heat damage. Hence in our study, IR64, HT NIL in IR64 background, and the known heat-tolerant *aus* type N22 (Jagadish *et al.*, 2010), together with two hybrids, were exposed to post-flowering heat stress with the following objectives: (i) to compare the differential impact of HDT, HNT, and HNDT on parameters related to grain growth and development; (ii) to test if the known HDT-tolerant NIL in IR64 background has a positive influence on maintaining grain quality under stress; and (iii) to determine the impact of HNT, HDT, and HNDT on key starch metabolizing enzymes and their influence on starch packaging in developing rice grains.

## Materials and methods

### Plant material and experimental set-up

Five rice genotypes were used: Nagina 22 (N22; heat tolerant), IR64 (heat susceptible), heat tolerant IR64 near-isogenic line (HT NIL) (Ye *et al.*, 2012), and two hybrids, H2 (private company hybrid) and H5 (International Rice Research Institute hybrid breeding programme) (the numbering of the hybrids is based on Shi *et al.* (2016) for ease of comparison across studies). The two hybrids were selected based on their higher relative difference in seed-set (H2) and grain weight (H5) under HNT exposure (Shi *et al.*, 2016) and also to represent private and public breeding products.

Dormancy of the seeds was broken by exposing seeds to 50 °C for 3 d, and pre-soaked seeds were sown in seeding trays. One 14-day-old seedling was transplanted into a 7-liter plastic pot (23 cm diameter and 25 cm height) containing 6 kg clay loam soil. Basal fertilizer of 2.0 g ammonium sulfate [(NH_4_)_2_SO_4_], 1.0 g single superphosphate, and 1.0 g muriate of potash (KCl) was applied to each pot. An additional 2.0 g (NH_4_)_2_SO_4_ was top-dressed at 25 d after transplanting. The study was conducted at the International Rice Research Institute, Los Baños, Philippines (14°11′N, 121°15′E, 21 m asl). Plants were grown in pots in a naturally lit greenhouse until flowering, and were then moved to controlled-environment walk-in chambers where plants were subject to various temperature treatments (see next section). All pots were maintained under flooded condition from transplanting to harvest to avoid water stress. No major pests and diseases were noticed during the experiment.

### Temperature treatments and growth chamber conditions

At the onset of flowering of the main and/or primary tillers from each plant, the flowering spikelets from the top portion of the panicle (located on upper primary rachis branches) were marked (i) to collect samples of developing grains, temporally, having the day of flowering as the common reference across genotypes and treatments and (ii) to avoid collecting samples that would confound findings due to the known gradient in grain developmental differences from the top (superior spikelets) towards the bottom (inferior spikelets) portion of a panicle (Yang and Zhang, 2010). Use of only the superior spikelets will allow the testing of whether assimilate supply is the major factor leading to lower single-grain weight and poor quality under exposure to heat stress. The following day after marking, 50 pots (one plant per pot) per genotype per temperature treatment were moved into large walk-in growth chambers (3.3 m×3.2 m×2.7 m; 10.6 m^2^ area) programmed to induce temperature treatments. The temperature treatments were randomly assigned to four independent chambers and plants were randomly arranged in a chamber. Each chamber was fitted with six independent units of 1 kW high-intensity discharge lamps, providing photosynthetic photon flux density of ≥650 μmol m^−2^ s^−1^ at plant canopy for 11 h and 215 μmol m^−2^ s^−1^ for 2 h during 05.00–06.00 h and 17.00–18.00 h, providing a total photoperiod of 13 h day^−1^. Relative humidity (RH) in the chambers was set at 70%. Plants were exposed to control temperature (Control, day/night, 31 °C/23 °C), high day-time temperature (HDT, 38 °C/23 °C), high night-time temperature (HNT, 31 °C/30 °C), and combined high night-time and day-time temperatures (HNDT, 38 °C/30 °C). The HDT temperature of 38 °C was maintained from 08.30 h to 14.30 h for 6 h while the HNT temperature of 30 °C was for 11 h from 18.00 h to 05.00 h (in order to obtain short episodes of heat spikes during the day *versus* long period of warmer nights with less fluctuation, replicating field conditions in tropical rice growing regions). In addition, the night temperature as observed in our earlier studies did not follow a sinusoidal pattern as the day temperature did and the conditions were maintained relatively similar over the entire night, further supporting our temperature treatment structure. The other hours (14.30–18.00 and 05.00–08.30 h) in a diurnal cycle were the gradual temperature change-over periods. Temperature treatments were imposed for 20 consecutive days after flowering, a period identified to be determining grain weight and grain quality in rice (Gong *et al.*, 2013; Ishimaru *et al.*, 2003). For our experiment, these 20 d covered almost the entire grain-filling duration (see Results). After the treatment, the plants were transferred back into the greenhouse until physiological maturity under the natural condition where the temperature recorded during hours similar to the stress duration (08.30–14.30 h) were 31.5 °C (SD=1.6 °C) during day-time and 25.4 °C (SD=1.0 °C) at night-time (18.00–05.00 h). It took 5–8 d for N22 and 10–12 d for the other genotypes to reach physiological maturity after the stress was released. Temperature and RH were continuously monitored at 15-min intervals at plant level (about 1.3–1.5 m from the ground surface) inside the chambers by using a Micrometeorological Instrument for Near Canopy Environment of Rice (MINCER, developed by the National Institute of Agrobiological Sciences, Japan; Yoshimoto *et al.*, 2012). All actual temperatures, RH and vapor pressure deficit (VPD) in all walk-in growth chambers during experiments are included in Supplementary Table S1 at *JXB* online. The VPD was calculated by using the equation presented in the website http://cronklab.wikidot.com/calculation-of-vapour-pressure-deficit.

### Grain development measurements

About 30–50 spikelets that flowered on the same day for each treatment were randomly collected at 2-day intervals until the end of the treatments (ten time points) and at physiological maturity for estimating multiple parameters that characterize grain filling. Instead of repeatedly sampling from the same set of plants, which is expected to generate a confounding effect on source–sink relationships, we sampled spikelets at various time points from independent replicate plants. All fertile spikelets that formed grains were counted and weighed after oven-drying at 70 °C until constant dry weight was reached. The observed single-grain dry weight (*W*) and days after flowering (*t*) were used to fit the determinate sigmoid growth equation as described in detail in Yin *et al.* (2003, 2009) and used in Shi *et al.* (2017), to describe the temporal dynamics of single-grain growth:

$$W=\{\begin{array}{ll} W_{\text{b}}+(W_{\text{max}}-W_{\text{b}})\left(1+\frac{t_{\text{e}}-t}{t_{\text{e}}-t_{\text{m}}}\right){\left(\frac{t-t_{\text{b}}}{t_{\text{e}}-t_{\text{b}}}\right)}^{\frac{t_{\text{e}}-t_{\text{b}}}{t_{\text{e}}-t_{\text{m}}}} & \;\text{if}\;t_{\text{b}}\leq t\leq t_{\text{e}}\\ W_{\text{max}} & \;\text{if}\;t>t_{\text{e}} \end{array}\;$$ (1)

where initial grain weight *W*_b_ is the grain weight at the time *t*_b_ when growth of grain begins, and *W*_max_ is the maximum value of single-grain weight that is achieved at the end of grain growth (*t*_e_). The mean grain-filling rate $\bar{C}$ is calculated from $\bar{C}$ =(*W*_max_−*W*_b_)/(*t*_e_−*t*_b_), while the maximum grain-filling rate *C*_m_, which is achieved at the time of the maximum growth rate (*t*_m_), is calculated by

$$\;C_{\text{m}}=(W_{\text{max}}-W_{\text{b}})\left[\frac{2t_{\text{e}}-t_{\text{m}}-t_{\text{b}}}{\left(t_{\text{e}}-t_{\text{b}})(t_{\text{e}}-t_{\text{m}}\right)}\right]{\left(\frac{t_{\text{m}}-t_{\text{b}}}{t_{\text{e}}-t_{\text{b}}}\right)}^{\frac{t_{\text{m}}-t_{\text{b}}}{t_{\text{e}}-t_{\text{m}}}}$$ (2)

At physiological maturity, the final set of marked grains were collected and evaluated individually. Partially filled grains with incomplete grain filling (see Fig. 1F in Shi *et al.*, 2015), and filled and unfilled grains were counted separately. Seed-set was determined by the number of fully filled and half-filled grains divided by the total number of marked grains. Dry weight of filled grains was obtained after oven-drying at 70 °C for 3 d.

### Enzyme assays and biochemical characterization

Grains at 5, 10, and 15 d after flowering (DAF) were collected consistently at the same time across sampling dates. Specifically, samples of grains for HNT and those for the respective control were collected at 04.00 h (i.e. towards the end of the night-time stress exposure), while for HDT, HNDT and control treatments, grain samples were collected at 14.00 h (i.e. towards the end of the day-time treatment). The samples were immediately submerged in liquid nitrogen and stored at –80 °C for subsequent enzyme assays. Activities of four key enzymes involved in sucrose-to-starch conversion (cell wall invertase, vacuolar invertase, sucrose synthase and soluble starch synthase) were determined. All chemicals and enzymes used for enzyme estimation were from Sigma-Aldrich (St Louis, MO, USA). We followed exactly the same protocol for enzyme extraction and activity assay as described in Bahuguna *et al.* (2017). Enzyme activity was expressed in nanomoles per milligram protein per hour for sucrose synthase and nanomoles per milligram protein per minute for others (for details, see Bahuguna *et al.*, 2017).

Grains at 5, 10, and 15 DAF and at physiological maturity were obtained to assess non-structural carbohydrates (NSC) content. Briefly, 0.1 g of finely ground grain samples were extracted with 7 ml of ethanol (80% v/v) at 85 °C for 10 min and repeated three times. The supernatant was transferred after centrifugation and total volume was adjusted to 25 ml by combining all supernatants from washed pellet and also the 80% ethanol. Then soluble sugar content was measured by using anthrone reagent as described in Yoshida *et al.* (1976). The remaining residue was dried in an oven for 24 h. Then, 2 ml of water was added into the dried residue before placing the tubes in a boiling-water bath for 15 min. After cooling on ice, 2 ml of 9.2 N HClO_4_ was added and the tubes were stirred occasionally for 15 min. The suspension was adjusted to 6 ml by adding water and then the supernatant was transferred after centrifugation. These steps were repeated by adding 2 ml of 4.6 N HClO_4_ and water to wash the residue, respectively. All supernatants were combined and the total volume was adjusted to 50 ml with water. The starch content was read by a colorimetric method with anthrone reagent at 630 nm (Yoshida *et al.*, 1976).

### Observation of chalkiness

To observe the endosperm structure of the developing grains, grains were collected at 5, 10, and 15 DAF for each of the four treatments and were carefully divided into halves by using the edge of a sharp razor blade to create natural fracture surfaces (Cao *et al.*, 2016) to obtain a cross section of the grains. The separated halves were fixed on aluminium specimen stubs using a double-sided tape, and the specimen’s surface was coated with gold using an ion sputtering device (JFC-1100E, JEOL, Tokyo, Japan) under vacuum. Then the samples were observed and photographed with a scanning electron microscope (XL-30, Philips, The Netherlands). At maturity, fully matured grains were collected for evaluation of grain appearance: the hulled grains were viewed visually and classified into transparent, milky-white/white-cored, white-belly, and opaque kernels according to the classification of Tsukaguchi and Iida (2008).

### Statistical analysis

The data obtained for seed-set, single-grain weight, enzyme activities, NSC content and chalkiness were analyzed as a completely randomized design following ANOVA using GenStat 16ED (Rothamsted Experimental Station, Harpenden, UK), and the mean values were compared based on the least significant difference (LSD) test at a 5% probability level. The curve fitting of Eqn 1 was carried out using least-squares non-linear regression with the GAUSS method in PROCNLIN of SAS (SAS Institute Inc., Cary, NC, USA), and mean and maximum grain-filling rates ( $\bar{C}$ and *C*_m_) were calculated thereof.

## Results

### Seed-set

A significant genotype×treatment (*P*<0.001) effect was recorded for seed-set based on the marked spikelets (Table 1). There was a significant reduction in the seed-set percentage under HNT exposure only in IR64 (7.7%), while HT NIL and both hybrids behaved similarly to the heat tolerant N22. In contrast, seed-set was significantly reduced in all genotypes with HDT except in HT NIL, with least reduction in HT NIL (3%) and the highest in IR64 (17%). HNDT exposure led to significant reduction in seed-set across all tested genotypes compared with the control, with least reduction in H5 and N22 (5–6%) while the other three genotypes recorded reductions of 10–12%. To test the relative importance of day-time temperature (*T*_day_) and night-time temperature (*T*_night_), as well as their interaction (*T*_day_×*T*_night_), regression analysis was carried out and the results are included in Supplementary Tables S2 and S3. Across five genotypes, *T*_day_ was more damaging than *T*_night_ for seed-set as absolute values of the negative coefficients of *T*_day_ were larger than those of *T*_night_ (Supplementary Table S2). Besides, the relative impact of *T*_day_ over *T*_night_ depended on genotype, there was a tendency for the difference between the coefficients of *T*_day_ and *T*_night_ to be smaller in HT NIL than in the other genotypes, suggesting the difference in sensitivity to *T*_day_ and *T*_night_ was smaller in the heat-tolerant NIL genotype. On the other hand, IR64 and H5 showed a significant *T*_day_×*T*_night_ interaction (*P*<0.05) for seed-set (Supplementary Table S3).

**Table 1.** Seed-set and single-grain weight of filled grains of five rice genotypes exposed to control temperature (31 °C/23 °C, day/night), high night-time temperature (HNT; 31 °C/30 °C), high day-time temperature (HDT; 38 °C/23 °C), or combined high night-time and day-time temperature (HNDT; 38 °C/30 °C) during grain filling for 20 consecutive days. The values are the mean±standard error of the mean and least significant difference; ***significant at the 0.001 probability level.

	Seed-set (%)				Single-grain weight (mg grain^−1^)			
Genotype	Control	HNT	HDT	HNDT	Control	HNT	HDT	HNDT
N22	96.3 ± 0.7	95.2 ± 0.9	88.8 ± 2.9	90.9 ± 1.7	17.1 ± 0.2	17.2 ± 0.2	15.0 ± 0.6	12.9 ± 0.6
IR64	94.9 ± 1.2	87.6 ± 1.3	79.1 ± 2.8	83.3 ± 1.7	23.3 ± 0.1	23.4 ± 0.1	17.5 ± 0.6	14.2 ± 0.3
HT NIL	94.5 ± 1.1	92.3 ± 1.0	91.2 ± 1.8	84.8 ± 4.6	25.2 ± 0.2	25.0 ± 0.1	21.2 ± 0.6	20.2 ± 0.9
H2	92.6 ± 1.6	92.3 ± 1.3	87.9 ± 1.7	82.7 ± 1.7	20.6 ± 0.2	20.5 ± 0.5	17.5 ± 0.3	13.4 ± 0.3
H5	88.4 ± 1.0	86.5 ± 0.9	78.6 ± 2.0	83.9 ± 1.8	22.3 ± 0.2	22.2 ± 0.3	20.7 ± 0.2	17.7 ± 0.4
Genotype	1.9***				0.3***			
Treatment	1.7***				0.3***			
Genotype×Treatment	3.7***				0.7***			

### Grain-filling parameters and single-grain weight

We used Eqn 1 to fit data on the time course of grain filling. In line with the use of Eqn 1 by Yin *et al.* (2009), we set flowering as the starting reference point, i.e. set *t*_b_=0 as the onset time of grain filling, and let the model fit parameters *W*_b_, *W*_max_, *t*_m_, and *t*_e_. Variations in grain-filling parameters were effectively estimated using the model (*R*^2^=0.93–0.99) across all genotypes and treatments (Table 2), as confirmed by the result that the estimated *W*_max_ values (Table 2) were essentially the same as the observed mean grain weight (Table 1). Using these estimates, maximum (*C*_m_, Eqn 2) and mean grain-filling rates ( $\bar{C}$ ) were calculated (Table 2).

**Table 2.** *Grain-filling parameter values (standard error of the estimate in parentheses) derived through Eqn 1 for five rice genotypes exposed to control (31 °C/23 °C, day/night), high night-time temperature (HNT; 31 °C/30 °C), high day-time temperature (HDT; 38 °C/23 °C) or combined high night-time and day-time temperature (HNDT; 38 °C/30 °C) at grain filling for 20 d after flowering*. $\bar{C}$ , mean grain-filling rate; *C*_m_, maximum grain-filling rate; *t*_e_, time at which the maximum grain weight is reached; *t*_m_, time when the maximum growth rate is achieved; *W*_b_, initial grain weight; *W*_max_, maximum value of grain weight.

Genotype	Treatment	*W*_max_ (mg grain^−1^)	*W*_b_ (mg grain^−1^)	*t*_m_ (day)	*t*_e_ (day)	*C*_m_ (mg grain^−1^ day^−1^)	$\bar{C}$ (mg grain^−1^ day^−1^)	*R* ^2^
N22	Control	16.78 (0.17)	2.32 (0.36)	7.68 (0.28)	12.33 (0.41)	1.96	1.17	0.98
	HNT	16.74 (0.14)	2.20 (0.37)	5.89 (0.32)	11.13 (0.36)	2.00	1.31	0.99
	HDT	14.27 (0.20)	2.27 (0.43)	7.11 (0.39)	11.59 (0.52)	1.71	1.04	0.97
	HNDT	13.10 (0.22)	2.43 (0.59)	5.87 (0.56)	10.15 (0.77)	1.67	1.05	0.93
IR64	Control	23.73 (0.68)	2.09 (0.75)	8.11 (1.37)	23.62 (1.61)	1.32	0.92	0.97
	HNT	23.10 (0.56)	1.94 (0.72)	6.10 (1.44)	22.64 (1.48)	1.37	0.93	0.97
	HDT	16.42 (0.35)	2.87 (0.36)	9.93 (0.68)	20.74 (1.07)	0.97	0.65	0.98
	HNDT	14.41 (0.23)	2.82 (0.52)	7.23 (0.65)	13.07 (0.85)	1.38	0.89	0.95
HT NIL	Control	24.23 (0.45)	2.21 (0.45)	10.03 (0.53)	20.84 (0.84)	1.57	1.06	0.99
	HNT	24.40 (0.58)	1.05 (1.18)	4.75 (1.86)	19.10 (1.56)	1.80	1.22	0.96
	HDT	21.56 (0.28)	2.78 (0.39)	9.53 (0.40)	17.66 (0.62)	1.64	1.06	0.99
	HNDT	21.15 (0.20)	3.24 (0.45)	7.45 (0.34)	13.05 (0.48)	2.17	1.37	0.98
H2	Control	20.10 (0.29)	2.20 (0.39)	8.90 (0.54)	19.29 (0.75)	1.37	0.93	0.99
	HNT	20.51 (0.24)	2.08 (0.50)	6.59 (0.70)	16.83 (0.72)	1.58	1.10	0.98
	HDT	17.28 (0.34)	2.26 (0.47)	9.26 (0.68)	18.35 (0.99)	1.23	0.82	0.97
	HNDT	14.62 (0.23)	2.62 (0.51)	6.86 (0.47)	11.39 (0.60)	1.72	1.05	0.95
H5	Control	21.99 (0.43)	2.23 (0.50)	9.92 (0.57)	19.52 (0.90)	1.53	1.01	0.98
	HNT	21.99 (0.23)	2.02 (0.49)	7.32 (0.43)	14.57 (0.52)	2.06	1.37	0.99
	HDT	19.21 (0.35)	2.94 (0.49)	10.43 (0.49)	17.10 (0.80)	1.57	0.95	0.98
	HNDT	17.18 (0.15)	2.55 (0.32)	7.38 (0.34)	13.78 (0.45)	1.63	1.06	0.99

Across all genotypes, the maximum (*C*_m_) and mean ( $\bar{C}$ ) grain-filling rates were higher with HNT than for the control, whereas the time taken to reach the maximum grain-filling rate (*t*_m_) and total grain-filling duration (*t*_e_) were shortened by HNT compared with the control. Thus, the combination of an increased grain-filling rate ( $\bar{C}$ increased by 1.1–35.6%) and shortened total grain-filling duration (*t*_e_ decreased by 4.1–25.4%) did not result in a lower final single-grain weight under HNT compared with the control condition (Table 1), indicating compensation of reduced grain-filling duration by increased rate of filling. Comparatively, the values of *C*_m_ and $\bar{C}$ of the five genotypes were largely decreased with HDT compared with the control treatment, except for an increase in *C*_m_ in HT NIL and H5 (Table 2), as well as no change in $\bar{C}$ of HT NIL. Additionally, HDT reduced grain-filling duration (*t*_e_) compared with the control conditions, which resulted in the reduction of single-grain weight in all genotypes (Table 1).

**Fig. 1.**
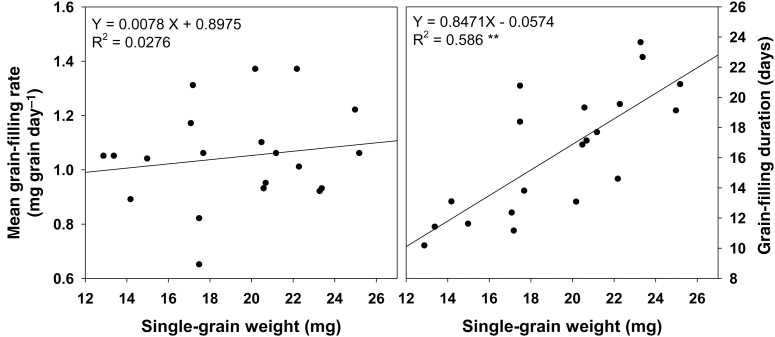
The relationship of mean grain-filling rate or grain-filling duration (*t*_e_, the time to reach maximum grain weight) and final single-grain weight across five genotypes grown at control (31 °C/23 °C, day/night), high night-time temperature (HNT; 31 °C/30 °C), high day-time temperature (HDT; 38 °C/23 °C) or combined high night-time and day-time temperature (HNDT; 38 °C/30 °C) at grain filling lasting for 20 d after flowering.

Four out of the five genotypes recorded higher *C*_m_ upon exposure to HNDT compared with control conditions; the exception was N22. The mean grain-filling rate ( $\bar{C}$ ) was decreased in N22 and IR64, with an increase in H5 and H2 and a very strong increase in HT NIL. Across all five genotypes, time taken to reach the maximum grain-filling rate and grain-filling duration were largely shortened under HNDT compared with control. Moreover, there was a strong reduction in total grain-filling duration of all genotypes under HNDT (21.3–37.1%) compared with a smaller and more variable reduction with HNT (4.1–25.4%) and a similar reduction under HDT (4.9–15.3%) exposure. Therefore, the final single-grain weight under HNDT was the lowest compared with other treatments and the impact was the same for all five genotypes (Table 1). Single-grain weight was significantly and positively correlated with total grain-filling duration (*t*_e_), with a non-significant positive relationship between single-grain weight and mean grain-filling rate (Fig. 1).

Regarding the *T*_day_ and *T*_night_ effects on single-grain weight, *T*_day_ had greater impact than *T*_night_ (Supplementary Table S2). Even though four out of five genotypes (except HT NIL) had significant interaction between *T*_day_ and *T*_night_, the interaction was significant at a very high probability level for single-grain weight (*P*<0.001, Supplementary Table S3). This suggests that although *T*_day_ was dominant, *T*_night_ interacts with *T*_day_ in determining grain weight, in all tested genotypes except HT NIL.

### Sink-related enzymatic activity

**Fig. 2.**
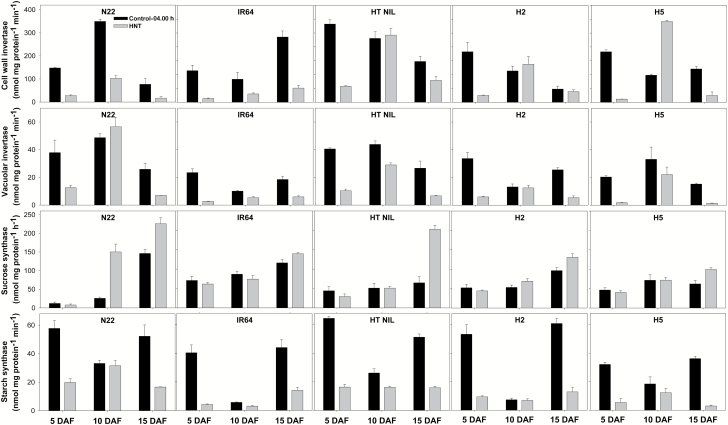
Cell wall invertase, vacuolar invertase, sucrose synthase and starch synthase in grains taken at 04.00 h on 5, 10, and 15 d after flowering (DAF) in five rice genotypes exposed to control (31 °C/23 °C, day/night) and high night-time temperature (HNT; 31 °C/30 °C) at grain-filling stage lasting for 20 d after flowering.

A significant genotype×treatment×stage effect (*P*<0.001) was observed for cell wall invertase (CWI) activity of grains taken from control and HNT at 04.00 h (Supplementary Table S4). Under HNT exposure, CWI activity was significantly reduced across genotypes and stages, except for non-significant changes in HT NIL at 10 DAF, and H2 at both 10 and 15 DAF, and a significant increase in H5 at 10 DAF (Fig. 2). Although not significant, a similar increasing trend in CWI was seen in HT NIL and H2 at 10 DAF. A significant genotype×stage (*P*<0.001) and treatment×stage (*P*<0.001) effect was observed for vacuolar invertase (VI) activity. HNT reduced the VI activity in the grains with the highest reduction recorded at 5 (61% to 91%) and 15 (68% to 92%) DAF, but there was less reduction at 10 DAF (5% to 47%) or even an increase in N22. For sucrose synthase (SuSy) activity, a significant genotype×treatment×stage effect (*P*<0.001) was observed. HNT did not induce significant changes in the grains at 5 DAF and 10 DAF except for N22 at 10 DAF. In contrast, SuSy activity was significantly increased in the grains sampled at 15 DAF for all genotypes under HNT exposure, with HT NIL recording the highest increase (214%). Significant genotype×treatment (*P*<0.05), genotype×stage (*P*<0.001) and treatment×stage (*P*<0.001) effects were observed for starch synthase (SS) activity. HNT significantly decreased SS activity across all five genotypes and at three different grain growth stages, except for N22, IR64, and H2 at 10 DAF. Moreover, the largest reduction was observed at 5 DAF (66–90%) and 15 DAF (68–91%) in SS activity in all genotypes while there was only a reduction of 5–47% at 10 DAF.

**Fig. 3.**
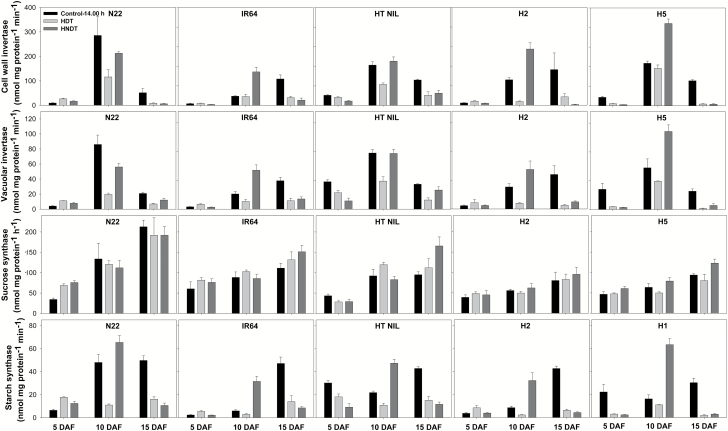
Cell wall invertase, vacuolar invertase, sucrose synthase, and starch synthase in grains taken at 14.00 h on 5, 10, and 15 d after flowering (DAF) in five rice genotypes exposed to control (31 °C/23 °C, day/night), high day-time temperature (HDT; 38 °C/23 °C) or combined high night-time and day-time temperature (HNDT; 38 °C/30 °C) at grain-filling stage lasting for 20 d after flowering.

A significant genotype×treatment×stage effect (*P*<0.001) was observed for CWI activity of the grains taken from control, HDT, and HNDT treatments at 14.00 h (Supplementary Table S4). Grains had lower CWI activity at 5 DAF, which was further reduced when they were exposed to HDT and HNDT compared with the control, except for N22, IR64, and H2 with relatively higher CWI activity at HDT compared with the control (Fig. 3). Furthermore, the reduction in CWI activity under both HDT and HNDT at 15 DAF was highly significant and consistent across all five genotypes. In comparison, CWI activity at 10 DAF was decreased under HDT compared with the control treatment while it increased under HNDT except for genotype IR64. A significant genotype×treatment×stage effect (*P*<0.001) was also observed for VI activity in the grains sampled from control, HDT, and HNDT treatments at 14.00 h. HDT induced a reduction in VI activity across all genotypes and three stages except for N22, IR64, and H2 at 5 DAF. In contrast, HDNT reduced VI activity at 5 and 15 DAF while there was an increase in VI activity at 10 DAF under HNDT compared with control in all genotypes except N22. A significant genotype×stage effect (*P*<0.001) was recorded for SuSy activity of the grains sampled from control, HDT, and HNDT treatments at 14.00 h (Supplementary Table S4). Thus, changes in SuSy activity depended on genotype in both HDT and HNDT conditions, but its activity tended to increase when grain growth progressed. For SS activity, a significant genotype×treatment×stage effect (*P*<0.001) was also observed for grains sampled from control, HDT, and HNDT treatments at 14.00 h. Under HDT, SS activity was significantly reduced across all genotypes and three grain-growth stages except for a slight increase in N22, IR64, and H2 at 5 DAF. In contrast, the activity of SS was significantly lower at 5 and 15 DAF when exposed to HNDT although no significant changes were recorded in N22, IR64, and H2 at 5 DAF, while its activity at 10 DAF was significantly higher across all genotypes under HNDT exposure.

### Content of non-structural carbohydrates

**Fig. 4.**
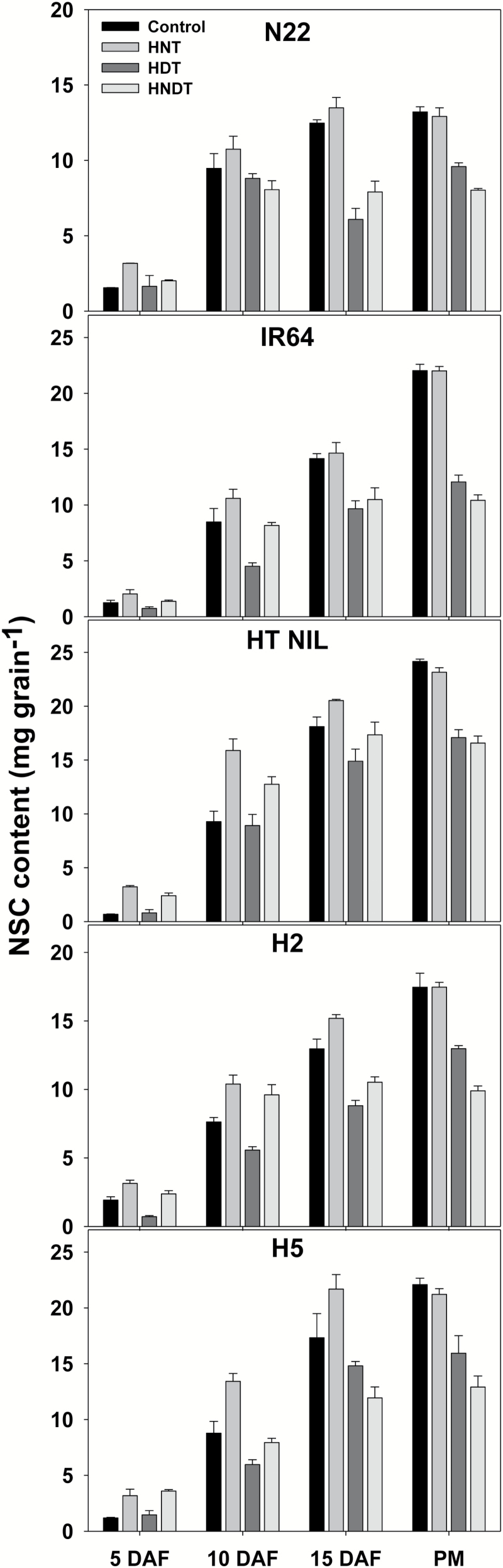
Non-structural carbohydrates (NSC) content in grains on 5, 10, and 15 d after flowering (DAF) and at physiological maturity (PM) in five rice genotypes exposed to control (31 °C/23 °C, day/night), high night-time temperature (HNT; 31 °C/30 °C), high day-time temperature (HDT; 38 °C/23 °C) or combined high night-time and day-time temperature (HNDT; 38 °C/30 °C) at grain-filling stage for 20 d after flowering. ANOVA results (values are least significant difference following by the significance level; ***P<0.001): genotype (G), 0.44***; treatment (T), 0.39***; stage (S), 0.39***; G×T, 0.88***; G×S, 0.88***; T×S, 0.79***; G×T×S, 1.76***.

A significant genotype×treatment×stage effect was observed for the NSC content in the grains (Fig. 4). The faster grain-filling rate of grains when exposed to HNT was supported by higher NSC content with grains exposed to HNT than with grains that developed under control conditions in all genotypes at 5, 10, and 15 DAF, with NSC content under HNT exposure being close to control treatment at final maturity. Under HDT conditions, the NSC content did not change significantly in all genotypes at 5 DAF while it was significantly lower than under control conditions at 10 DAF, 15 DAF, and maturity across all genotypes except for the non-significant effect in N22 and HT NIL at 10 DAF. For the HNDT treatment, differences among genotypes and stages were observed in the NSC content. In all genotypes, grains grown under HNDT showed higher NSC content than those grown under control conditions at 5 DAF. At 10 DAF, significantly higher NSC content was only observed in HT NIL and H2, while the other genotypes had lower NSC content than the control. After that (at 15 DAF and maturity), the NSC content under HNDT was significantly lower than for the control in all five genotypes.

### Development of amyloplasts

**Fig. 5.**
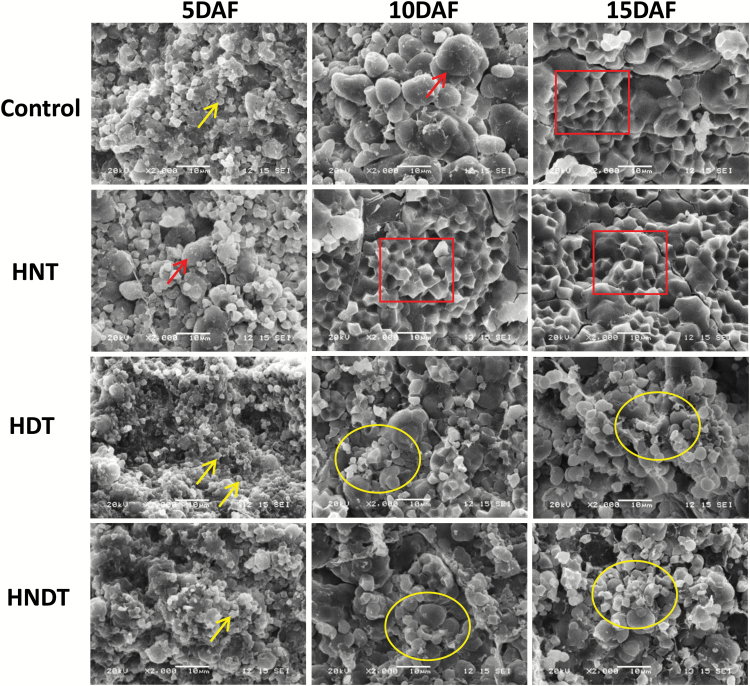
Scanning electron microscopy observation of the transverse section of the central part of the developing grains collected at 5, 10, and 15 d after flowering (DAF) in IR64 exposed to control (31 °C/23 °C, day/night), high night-time temperature (HNT; 31 °C/30 °C), high day-time temperature (HDT; 38 °C/23 °C) or combined high night-time and day-time temperature (HNDT; 38 °C/30 °C) at grain-filling stage for 20 d after flowering. Scale bars: 10 µm. Arrows for 5 DAF control, HDT, and HNDT indicate single granules. Arrows for 5 DAF HNT and 10 DAF control indicate single granules grouping into amyloplast. Rectangles indicate the polygonal shape of starch granules grouping into amyloplast without airspaces. Elliptical rings shows poorly developed amyloplasts together with the individual round shape and heterogeneous size of starch granules with large airspaces. (This figure is available in color at *JXB* online.)

To understand the effects of heat stress on the development of the endosperm, transverse sections of the central part of the grains were analysed under a scanning electron microscope (Fig. 5 and Supplementary Figs S1–S3). At 5 DAF, small starch granules had developed in the rice endosperm across all genotypes and four treatments. Besides, starch granules began packaging into amyloplasts, particularly in the grains exposed to HNT, indicating that the grain-filling process in this treatment was more advanced than in the control, HDT, and HNDT treatments while there were no obvious differences in the starch granules under both HDT and HNDT condition. Starting at 10 DAF, amyloplasts were compounded and tightly packed with numerous well-developed (polygonal shape) starch granules in developing grains under HNT exposure in all genotypes, while this phenomenon was only observed in N22 and H2 under control conditions (Supplementary Fig. S2). In contrast, the starch granules in grains exposed to HDT and HNDT were poorly developed (round shape together with heterogeneous size) and single, that is to say not all starch granules participated in the packing process towards amyloplast development. In addition, large airspaces were observed between amyloplasts or the individual starch granules in the grains exposed to HDT and HNDT. Thus, the scanning electron microscopy results illustrated poor development of starch granules in the grains exposed to HDT and HNDT conditions, which could have resulted in lower single-grain weight and poor grain quality, i.e. the formation of chalk.

### Chalkiness

To ascertain the heat-stress impacts on the occurrence of different types of chalky kernels, the grains harvested at maturity were hulled manually and assessed visually. The percentage of the various types of chalk kernel was examined per treatment for each genotype (Table 3). Many grains were found with a large chalky part around the core, indicating either milky-white or white-core chalkiness, and were grouped into one category. A significant genotype×treatment effect was observed for the different types of chalkiness. More than 84% of the control grains were grouped into the transparent type across the five genotypes. However, HNT treatment significantly increased the percentage of white-belly grains (31.8% to 67.0%) in all genotypes and significantly induced an increase in proportion of milky-white or white-core grains in N22 and H2. In contrast, HDT and HNDT substantially increased the chalkiness: the proportions of milky-white or white-core grains were suddenly increased up to 72.6–91.7% (*P*<0.001), which accounted for the largest proportion, and opaque (fully chalky) kernels went up (*P*<0.001) to be the second largest proportion under both HDT and HNDT conditions. In summary, all high-temperature treatments resulted in a significant increase in chalkiness of the grains, but with HNT mainly resulting in white-belly chalkiness while HDT and HNDT resulted in high proportions of milky white and/or white-core chalkiness.

**Table 3.** *Total number of grains used for chalkiness observation and the percentage of each type of chalk grain in five rice genotypes exposed to control (31 °C/23 °C, day/night), high night-time temperature (HNT; 31 °C/30 °C), high day-time temperature (HDT; 38 °C/23 °C), or combined high night-time and day-time temperature (HNDT; 38 °C/30 °C) at grain filling lasting 20 d after flowering*. Mean value±standard error of the mean, and least significant difference; ***significant at the 0.001 probability level.

Genotype	Treatment	No. of grains observed	Transparent grain (%)	Milky-white and white-core (%)	White-belly (%)	Opaque kernels (%)
N22	Control	106	92.8 ± 1.0	1.4 ± 1.4	5.2 ± 1.3	0.0 ± 0.0
	HNT	145	34.4 ± 2.8	16.5 ± 2.8	45.8 ± 4.5	0.0 ± 0.0
	HDT	143	0.7 ± 0.7	88.3 ± 3.1	0.0 ± 0.0	11.0 ± 2.8
	HNDT	137	0.7 ± 0.7	87.0 ± 1.5	0.0 ± 0.0	12.3 ± 1.2
IR64	Control	173	88.5 ± 1.2	7.5 ± 1.5	2.9 ± 0.5	0.0 ± 0.0
	HNT	183	59.6 ± 0.6	5.9 ± 2.2	31.8 ± 3.3	2.7 ± 0.5
	HDT	194	0.5 ± 0.5	90.7 ± 0.8	0.0 ± 0.0	8.8 ± 0.6
	HNDT	116	0.0 ± 0.0	84.5 ± 1.9	0.0 ± 0.0	15.5 ± 1.9
HT NIL	Control	144	86.5 ± 2.1	0.0 ± 0.0	11.4 ± 2.0	1.5 ± 0.7
	HNT	257	40.4 ± 2.6	5.4 ± 3.0	50.3 ± 4.2	3.9 ± 1.1
	HDT	254	0.4 ± 0.4	90.5 ± 1.6	0.0 ± 0.0	5.8 ± 1.1
	HNDT	150	1.3 ± 1.3	89.2 ± 2.5	0.0 ± 0.0	9.5 ± 1.4
H2	Control	147	84.9 ± 4.5	1.2 ± 1.2	13.2 ± 3.7	0.0 ± 0.0
	HNT	185	38.3 ± 7.7	16.5 ± 6.7	43.2 ± 2.8	0.7 ± 0.7
	HDT	283	12.5 ± 1.6	72.6 ± 1.4	12.4 ± 2.3	1.0 ± 0.6
	HNDT	155	0.0 ± 0.0	91.7 ± 1.3	0.0 ± 0.0	8.3 ± 1.3
H5	Control	140	85.0 ± 0.4	5.0 ± 0.1	10.0 ± 0.3	0.0 ± 0.0
	HNT	224	30.3 ± 0.9	2.7 ± 0.8	67.0 ± 1.1	0.0 ± 0.0
	HDT	217	0.0 ± 0.0	73.4 ± 0.8	23.8 ± 1.1	2.8 ± 0.3
	HNDT	147	1.1 ± 1.1	78.4 ± 1.0	11.2 ± 0.8	9.4 ± 1.2
Genotype			3.4***	3.3***	3.0***	1.5***
Treatment			3.1***	3.0***	2.7***	1.4***
Genotype×Treatment			6.9***	6.7***	6.0***	3.1***

## Discussion

A comprehensive geographical mapping exercise on a global scale has indicated regions with higher vulnerability to HNT or HDT or a combined HNDT stress (Laborte *et al.*, 2012), substantiating the need for systematic investigation of the response of rice genotypes to these conditions. HNT of 30 °C coinciding with the time after flowering and maintained for a few additional days did not affect seed-set/spikelet fertility negatively under growth chamber and field conditions (Shi *et al.*, 2013; Jagadish *et al.*, 2015), which is supported by our findings. However, the present results indicate clear differential responses of rice genotypes to HDT and HNT, with a greater impact of HDT on seed-set compared with HNT (Supplementary Tables S2 and S3). This variable response was noted despite the shorter duration of increased day-time temperature per day imposed in our treatments (6 h for day-time treatment and 11 h for night-time treatment). The findings are in agreement with the results of Yin *et al.* (1996), showing that *T*_day_ exerts a greater influence on rice plant development than *T*_night_. Since the stress was imposed a day after anthesis, key physiological processes such as anther dehiscence and pollen germination would not be the primary determinants reducing seed-set (Jagadish *et al.*, 2007). Hence, the reduced seed-set would have mainly resulted from the impact on the embryo development, including division of the fertilized egg or primary endosperm nucleus and subsequent processes (Vara Prasad *et al.*, 2017). Since regulation of cell division, endo-reduplication and cell expansion varies during day and night, e.g. cell division is known to be stimulated by light (Okello *et al.*, 2015), the day-time temperature is more important in determining seed-set than the night-time temperature.

Similar to the seed-set, *T*_day_ induced greater damage than *T*_night_ for grain-growth patterns, whereas *T*_night_ interacted with *T*_day_ to determine single-grain weight. Previous studies involving single genotype in which night-time temperatures were extremely high (34 and 35 °C) together with relatively low day-time warming (34 and 35 °C) suggested that HNT has a larger negative impact on single-grain weight than HDT (Morita *et al.*, 2005; Li *et al.*, 2011). In contrast, day-time warming had greater effects on grain weight than night-time warming normalized by every 1 °C warming (Rehmani *et al.*, 2014), which is supported by our results. However, with the predicted increase in night-time temperature at a faster rate than day-time temperature, the negative influence of HNT on overall yield losses should not be underestimated (Shi *et al.*, 2013, 2016, 2017). At the whole plant level, we have demonstrated that HNT of 29 °C starting from panicle initiation to maturity using field-based tents significantly reduces grain weight (Shi *et al.*, 2013, Jagadish *et al.*, 2015). Differential responses for grains at different positions within a panicle exposed to high temperatures have been documented (Cao *et al.*, 2016; Fu *et al.*, 2016). No decline in the single-grain weight under HNT in the current study could be attributed to the measuring approach wherein only superior spikelets having greater access to assimilate were considered (Fu *et al.*, 2016 and references therein). This approach using only superior spikelets allowed a common reference for explicitly estimating enzymatic activity and starch packaging during grain filling and avoided other confounding factors. Having sufficient assimilates available with stress imposed after flowering allowed marked spikelets to exercise plasticity to minimize damage and enabled ascertaining if the impact was primarily due to stress and not source limitation. However this may not be the case at the whole plant level, where grain weight is determined by the source–sink relationships altered by the loss of essential carbohydrates to enhanced night respiration (Bahuguna *et al.*, 2017), curtailing the level of plasticity.

Grain weight is mainly determined by a balance between grain-filling rate and grain-filling duration. The ability of the grain to retain its weight under HNT indicates the plasticity expressed under sufficient resource availability, which in our studies was made possible by following a measuring approach that included just the superior spikelets. On the other hand, the impact of HDT or HNDT induces irreversible damage either during the embryo development (seed-set reduction) or grain-filling stage (reduced grain weight), indicating the need to take a genetic route to enhance tolerance to HDT. Kim *et al.* (2011) suggested an early termination of grain filling in temperate rice exposed to high temperature was not due to lack of assimilates but to loss of sink activity. In contrast, Kobata and Uemuki (2004) have attributed the impact of HDT during grain filling to limitations in assimilate supply. Interestingly, we noticed that the targeted tillers exposed to HDT and HNDT treatments produced new extra tillers during the grain-filling stage in most cases, indicating surplus assimilates from the leaf and/or the reserves stored in the culm and leaf sheath. Thus, in our study the failure of assimilate supply to the grain was not the reason behind the lower grain weight under HDT and HNDT conditions; instead, the sink itself was playing a more important role, supporting Kim *et al.* (2011) in emphasizing the need to focus on sink strength under heat stress exposure.

The determination of dynamic grain growth in rice and other cereals has been related to the senescence of source and/or sink organs, i.e. loss of photosynthetic activity in the leaves and sugar or starch metabolism-related enzyme activity in the endosperm (Bahuguna *et al.*, 2017). The sink strength of a developing grain is met by a balanced sucrose gradient from source to sink tissue and by cleaving sucrose into hexoses by invertases and SuSy (Hirose *et al.*, 2002; Koch, 2004). Key starch metabolism enzymes including CWI (responsible for phloem unloading and cleaving sucrose in the apoplast; Wang and Ruan, 2012), VI (sink initiation and expansion by supporting cell division during the pre-storage phase; Roitsch and González, 2004), SuSy (supplying the substrate, UDP-glucose/ADP-glucose, for starch synthesis; Li *et al.*, 2013) and SS for starch synthesis were shown to be affected differently among rice genotypes under HNT exposure (Bahuguna *et al.*, 2017). Under HNT exposure, with developing grain samples collected at 04.00 h, the activity of CWI, VI and SS decreased especially at 5 and 15 DAF while SuSy activity remained rather stable across the three time points. Interestingly, the CWI and VI levels remained similar to control at the peak grain-filling stage, i.e. 10 d post-flowering (Bahuguna *et al.*, 2017), only in HT NIL and N22, respectively, indicating possible alternative routes to continued cleavage of transported sucrose. This along with the significantly higher SuSy activity in N22 (10 and 15 DAF) and HT NIL (15 DAF) indicates that they may be better equipped for harsher night-time temperature exposure compared with the other three genotypes. The reduced enzymatic activity did not contribute to derailing NSC accumulation and grain weight under HNT at the single-grain level, as reflected by the correlation coefficients in Supplementary Table S5. Significant relationships between key starch metabolism enzymes and grain-filling rate and duration at 5 and 10 DAF in spikelets exposed to HNT and HDT, respectively (Supplementary Tables S5 and S6), provides mechanistic support for an initial increase in grain weight and NSC accumulation (Fig. 4) in the grains exposed to HNT compared with control. Specific interactions call for further systematic investigation to minimize stress damage, such as (i) strong and opposite relationships between mean grain-filling rate and time taken for reaching maximum grain weight (*t*_e_) with sucrose synthase at 5 DAF under HNT and complete disappearance with HDT and HNDT and (ii) a strong negative relationship with *t*_e_ at 15 DAF in spikelets exposed to HDT and HNDT. However, Bahuguna *et al.* (2017) have shown lower CWI and SuSy activity to play a decisive role in NSC accumulation and grain weight under HNT exposure at the whole plant level, which was seen only at 5 DAF, indicating a strong impact of the source–sink relationship with total grain-filling duration at the whole plant level compared with the single-grain level.

The total NSC in grains under HDT and HNDT exposure was lower than that under control at the single-grain level. This low NSC could be a result of the unloading of transportable sugar (lower CWI activity), poor substrate supply for starch synthesis (lower SuSy activity), and low starch synthesis at the late grain-filling stage. However, at the single-grain level, a clear correlation with NSC accumulation in grains and enzymatic activity was not observed. Interestingly across all the tested genotypes and almost all the four key enzymes, the enzyme activity was increased considerably with HNDT compared with HDT at the peak grain-filling phase of 10 DAF. This could be attributed to either an accelerated phenomenon due to higher temperature or a short term acclimation response with high night-time alternating with high day-time temperature stress, which could prove beneficial if sufficient assimilates become available (resilient source-related sucrose transporters). Hence, exploring the ability of the key starch metabolism enzymes to acclimatize to increasing day-time or night-time temperature is an interesting area for further research.

In our study, HDT and HNDT resulted in smaller starch granules during the grain-filling period. This led to loosely packed starch granular structure leading to a more chalky appearance and lower single-grain weight, as documented by Geigenberger (2011). Moreover, milky-white/white-cored chalk grains were substantially increased under HDT and HNDT exposure, which is known to reduce the economic value of the grains (Lyman *et al.*, 2013). However, this phenomenon was not observed in HNT exposure as our scanning electron microscopy observations were aimed at the chalkiness at the central part of the grains, which is the most serious problem. Although the central part of developing grains had tightly packed polygonal shaped starch granules (amyloplasts) under the HNT condition, white-belly chalk was noticed to significantly increase under HNT. These results are in agreement with the observations in previous studies, which showed less effect on chalkiness under HNT compared with HDT (Dai *et al.*, 2009; Li *et al.*, 2011). The formation of milky-white/white-cored chalk under heat stress is mainly attributed to reduced assimilates supply (as indicated above with a possible source enzymatic failure with HDT) being unable to meet the enhanced demand resulting from a higher grain-filling rate (Liu *et al.*, 2013). In addition, white-belly chalk often occurs at the late stage due to inefficient utilization of reserves (Xi *et al.*, 2014). Lisle *et al.* (2000) suggested that chalk formation is more likely related to utilization of carbon during sucrose-to-starch deposition within the developing grains than an insufficient assimilate supply. From our findings we see that this holds true with HNT under sufficient assimilate availability while there was a limitation with both supply and utilization aspects under HDT and HNDT. Hence, the above findings highlight the importance of exploring the efficiency of source–sink activity at different forms of heat stress exposure.

HT NIL performed differently from the other genotypes in many respects. When exposed to HDT, HT NIL was the only genotype without significant decrease in seed-set compared with the others (Table 1), indicating its true tolerance and providing evidence for its post-flowering heat stress tolerance in addition to tolerance during flowering (Ye *et al.*, 2012). When exposed to HNDT, HT NIL responded similarly to other genotypes, but with a lower reduction in seed-set and with the least decrease in single-grain weight. In addition, HT NIL was the only genotype that maintained mean grain filling rate ( $\bar{C})$ under HDT or had the largest increase in $\bar{C}$ under HNDT, presumably contributing to significantly higher NSC content at 10 DAF under HDT and HNDT, respectively, while most other genotypes recorded significant decline in NSC at 15 DAF and at maturity (Table 2 and Fig. 4). Although investigation of the starch metabolism enzymes did not show striking differences in its responses to HDT and HNDT compared with the other genotypes, HT NIL had relatively higher CWI, VI, and SS activity at the peak grain-filling period, i.e. 10 DAF under HDT and HNDT conditions (Fig. 3). This provides partial mechanistic support for HT NIL in IR64 background tolerating heat stress during both flowering and post-flowering stages, making it an ideal source for further detailed molecular analysis to develop genetic markers for introducing sustained long duration heat stress tolerance into current susceptible popular rice cultivars.

## Conclusions

The impact of HNT, HDT, and HNDT during grain filling on physiological, biochemical, and histological aspects related to grain growth was quantified in a contrasting set of rice inbreds and hybrids. HDT and HNDT had pronounced negative impact on the starch biosynthetic enzyme activity and also on the NSC content of the grains leading to structural changes in the starch granules resulting in increased milky-white/white-core chalk. However, HNT did not induce a reduction in single-grain weight and NSC content due to the dynamic compensation of higher grain-filling rate and shortened grain-filling duration. Interestingly, the HT NIL, developed to minimize the heat-stress impact at flowering, was seen to have an extended period of tolerance beyond flowering, resulting in reduced heat-stress impact during grain-filling phase. Comparatively, day-time temperature either independently or in combination with HNT had a strong negative impact on processes including the starch packing. These results provide a comprehensive understanding of the impact of high day-time or night-time temperature and their combination on grain growth and form a starting point for further elucidation of the complex mechanisms responsible for differential responses of day-time and night-time temperatures and diel warming on rice plants.

## Supplementary data

Supplementary data are available at *JXB* online.

Table S1. Actual temperature, relative humidity and vapor pressure deficit recorded in the walk-in growth chambers.

Table S2. Regression analysis between seed-set, single-grain weight and day-time and night-time temperatures.

Table S3. Regression analysis between seed-set, single-grain weight and day-time, night-time temperatures, and interaction of day-time and night-time temperature.

Table S4. Significance of probability for enzymatic activities in grains from five rice genotypes at 5, 10, and 15 d after flowering.

Table S5. Correlation coefficients of the activities of four enzymes taken at 04.00 h from control and HNT with final grain weight, non-structural carbohydrates and grain-filling parameters.

Table S6. Correlation coefficients of the activities of four enzymes taken at 14.00 h from control, HDT, and HNDT with final grain weight, non-structural carbohydrates and grain-filling parameters.

Fig. S1. Scanning electron microscopy observation of the central part of the developing grains collected at 5 d after flowering in four rice genotypes exposed to four day and night temperature treatments.

Fig. S2. Scanning electron microscopic observation of the central part of the developing grains collected at 10 d after flowering in four rice genotypes exposed to four day and night temperature treatments.

Fig. S3. Scanning electron microscopic observation of the central part of the developing grains collected at 15 d after flowering in four rice genotypes exposed to four day and night temperature treatments.

## Supplementary Material

supplementary_tables_S1_S6_Figures_S1_S3

## Abbreviations:

CWI: cell wall invertase
DAF: days after flowering
HDT: high day-time temperature
HNT: high night-time temperature
HNDT: combined high night-time and day-time temperature
HT NIL: heat-tolerant near-isogenic line
NSC: non-structural carbohydrates
RH: relative humidity
SS: starch synthase
SuSy: sucrose synthase
*T*_day_: day temperature
*T*_night_: night temperature
VI: vacuolar invertase
VPD: vapor pressure deficit.
