# Supplementary material for: High day- and night-time temperatures affect grain growth dynamics in contrasting rice genotypes
Source: J Exp Bot. 2017 Oct 9;68(18):5233–45. doi: 10.1093/jxb/erx344 (PMC5853565; doi:10.1093/jxb/erx344)

Supplementary Information

**Title - High day-time and night-time temperature affect grain growth dynamics in contrasting rice genotypes**

**Authors -** Wanju Shi, Xinyou Yin, Paul C. Struik, Celymar Solis, Fangming Xie, Ralf C. Schmidt, Min Huang, Yingbin Zou, Changrong Ye, Krishna Jagadish S.V.

1. Table S1. Actual temperature, relative humidity and vapour pressure deficit recorded in the walk-in growth chambers.
2. Table S2. Regression analysis between seed-set, single-grain weight and day-time and night-time temperatures.
3. Table S3. Regression analysis between seed-set, single-grain weight and day-time, night-time temperatures, and interaction of day-time and night-time temperature.
4. Table S4. Significance of probability for enzymatic activities in grains from five rice genotypes at 5, 10, 15 days after flowering
5. Table S5. Correlation coefficients of the activities of four enzymes taken at 4 a.m. from control and HNT with final grain weight, non-structural carbohydrates and grain-filling parameters.
6. Table S6. Correlation coefficients of the activities of four enzymes taken at 2 p.m. from control, HDT and HNDT with final grain weight, non-structural carbohydrates and grain-filling parameters.
7. Figure S1. Scanning electron microscopic observation of the central part of the developing grains collected at 5 days after flowering in four rice genotypes exposed to four day and night temperature treatments.
8. Figure S2. Scanning electron microscopic observation of the central part of the developing grains collected at 10 days after flowering in four rice genotypes exposed to four day and night temperature treatments.
9. Figure S3. Scanning electron microscopic observation of the central part of the developing grains collected at 15 days after flowering in four rice genotypes exposed to four day and night temperature treatments.

Supplementary Table S1. Actual temperature, relative humidity and vapour pressure deficit records within the walk-in growth chambers which were set at control (31 °C/23 °C (day/night)), higher night-time temperature (HNT-31 °C/30 °C), higher day-time temperature (HDT-38 °C/23 °C) or combined higher night-time and day-time temperature (HNDT-38 °C/30 °C) for exposing the treatments to rice plants. Mean ± standard deviation. Data for day-time was from 08:30 h – 14:30 h for 6 h while night-time was recorded for 11 h from 18:00 h – 05:00 h

| | Temperature (°C) | | Relative Humidity (%) | | Vapour pressure deficit (kPa) | |
|---|---|---|---|---|---|---|
| Treatment | Day-time | Night-time | Day-time | Night-time | Day-time | Night-time |
| Control | 30.9 ± 0.5 | 22.9 ± 0.3 | 68.2 ± 5.4 | 72.6 ± 7.6 | 1.4 ± 0.5 | 0.8 ± 0.2 |
| HNT | 31.0 ± 0.6 | 29.8 ± 0.4 | 61.9 ± 9.5 | 71.9 ± 6.7 | 1.5 ± 0.3 | 1.6 ± 0.2 |
| HDT | 37.8 ± 0.7 | 22.7 ± 0.6 | 61.1 ± 10.2 | 68.6 ± 5.8 | 2.0 ± 0.2 | 0.9 ± 0.3 |
| HNDT | 37.8 ± 0.6 | 30.1 ± 0.4 | 67.9 ± 8.9 | 66.0 ± 6.8 | 1.9 ± 0.2 | 1.3 ± 0.1 |

Supplementary Table S2. Regression analysis (Y = a + b1*Tday + b2*Tnight) was carried out for the seed-set and single-grain weight for five genotypes grown at control (31 °C/23 °C (day/night)), higher night-time temperature (HNT-31 °C/30 °C), higher day-time temperature (HDT-38 °C/23 °C) or combined higher night-time and day-time temperature (HNDT-38 °C/30 °C) at grain filling lasting for 20 days after flowering.

| | | | | Coefficients | | | Standard error | | | P-*value* | | |
|---|---|---|---|---|---|---|---|---|---|---|---|---|
| Trait | Genotype | No. of observations | $R^2$ | Intercept | Tday | Tnight | Intercept | Tday | Tnight | Intercept | Tday | Tnight |
| Seed-set | N22 | 104 | 0.15 | 121.16 | -0.82 | 0.00 | 7.37 | 0.20 | 0.19 | 0.00 | 0.00 | 1.00 |
| | IR64 | 145 | 0.12 | 132.27 | -1.25 | -0.12 | 12.65 | 0.28 | 0.28 | 0.00 | 0.00 | 0.67 |
| | HT NIL | 98 | 0.07 | 130.21 | -0.73 | -0.52 | 14.48 | 0.28 | 0.27 | 0.00 | 0.01 | 0.05 |
| | H2 | 128 | 0.10 | 134.63 | -0.93 | -0.52 | 12.46 | 0.29 | 0.27 | 0.00 | 0.00 | 0.05 |
| | H5 | 155 | 0.10 | 106.06 | -0.87 | 0.30 | 10.88 | 0.23 | 0.23 | 0.00 | 0.00 | 0.20 |
| Single-grain weight | N22 | 103 | 0.46 | 34.20 | -0.47 | -0.10 | 2.11 | 0.06 | 0.05 | 0.00 | 0.00 | 0.08 |
| | IR64 | 147 | 0.77 | 65.42 | -1.13 | -0.25 | 2.37 | 0.05 | 0.05 | 0.00 | 0.00 | 0.00 |
| | HT NIL | 112 | 0.58 | 46.43 | -0.63 | -0.06 | 3.05 | 0.06 | 0.06 | 0.00 | 0.00 | 0.25 |
| | H2 | 131 | 0.61 | 52.46 | -0.68 | -0.41 | 2.53 | 0.06 | 0.05 | 0.00 | 0.00 | 0.00 |
| | H5 | 152 | 0.42 | 43.10 | -0.46 | -0.24 | 2.21 | 0.05 | 0.05 | 0.00 | 0.00 | 0.00 |

Supplementary Table S3. Regression analysis (Y = a + b1*Tday + b2*Tnight + b12*Tday*Tnight) was done for the seed-set and grain weight for five genotypes grown at control (31 °C/23 °C (day/night)), higher night-time temperature (HNT-31 °C/30 °C), higher day-time temperature (HDT-38 °C/23 °C) or combined higher night-time and day-time temperature (HNDT-38 °C/30 °C) at grain filling lasting for 20 days after flowering.

| Trait | | | | Coefficients | | | | Standard error | | | | P-*value* | | | |
|---|---|---|---|---|---|---|---|---|---|---|---|---|---|---|---|
| | Genotype | No. of observations | $R^2$ | Intercept | Tday | Tnight | Tday*Tnight | Intercept | Tday | Tnight | Tday*Tnight | Intercept | Tday | Tnight | Tday*Tnight |
| Seed-set | N22 | 104 | 0.16 | 180.79 | -2.60 | -2.22 | 0.07 | 50.87 | 1.52 | 1.89 | 0.06 | 0.00 | 0.09 | 0.24 | 0.24 |
| | IR64 | 145 | 0.17 | 355.31 | -7.63 | -8.28 | 0.23 | 77.02 | 2.19 | 2.80 | 0.08 | 0.00 | 0.00 | 0.00 | 0.00 |
| | HT NIL | 98 | 0.08 | 53.64 | 1.55 | 2.39 | -0.09 | 71.75 | 2.10 | 2.69 | 0.08 | 0.46 | 0.46 | 0.38 | 0.28 |
| | H2 | 128 | 0.11 | 33.97 | 1.87 | 3.36 | -0.11 | 78.36 | 2.17 | 2.99 | 0.08 | 0.67 | 0.39 | 0.26 | 0.20 |
| | H5 | 155 | 0.13 | 243.97 | -4.81 | -4.86 | 0.15 | 62.59 | 1.78 | 2.32 | 0.07 | 0.00 | 0.01 | 0.04 | 0.03 |
| Single-grain weight | N22 | 103 | 0.50 | -6.73 | 0.75 | 1.43 | -0.05 | 14.11 | 0.42 | 0.52 | 0.02 | 0.63 | 0.08 | 0.01 | 0.00 |
| | IR64 | 147 | 0.81 | -1.05 | 0.77 | 2.18 | -0.07 | 13.70 | 0.39 | 0.50 | 0.01 | 0.94 | 0.05 | 0.00 | 0.00 |
| | HT NIL | 112 | 0.58 | 32.07 | -0.20 | 0.48 | -0.02 | 15.01 | 0.44 | 0.56 | 0.02 | 0.03 | 0.65 | 0.39 | 0.33 |
| | H2 | 131 | 0.67 | -21.20 | 1.37 | 2.43 | -0.08 | 14.75 | 0.41 | 0.56 | 0.02 | 0.15 | 0.00 | 0.00 | 0.00 |
| | H5 | 152 | 0.49 | -12.51 | 1.13 | 1.82 | -0.06 | 12.80 | 0.37 | 0.47 | 0.01 | 0.33 | 0.00 | 0.00 | 0.00 |

Supplementary Table S4. Significance of probability for enzymatic activities in grains from five rice genotypes on 5, 10 and 15 days after flowering. The analysis was done separately based on the sampling time (control grains versus HNT grains taken at 4 a.m.; control, HDT, HNDT collected at 2 p.m.). Values are least significant difference for each trait followed by the significance level: * P<0.05, *** P<0.001, ns non-significant

| Treatment | Interactions | Cell wall intertase | Vacuolar invertase | Sucrose synthase | Starch synthase |
|---|---|---|---|---|---|
| Control at 4 a.m. and HNT | Genotype | 9.48*** | 3.94*** | 10.78*** | 3.53*** |
| | Treatment | 6.00*** | 2.49*** | 6.82*** | 2.23*** |
| | Stage | 7.34*** | 3.05*** | 8.35*** | 2.73*** |
| | Genotype × Treatment | 13.41*** | ns | 15.25*** | 4.99* |
| | Genotype × Stage | 16.42*** | 6.83*** | 18.67*** | 6.11*** |
| | Treatment × Stage | 10.39*** | 4.32*** | 11.81*** | 3.86*** |
| | Genotype × Treatment × Stage | 23.23*** | ns | 26.41*** | ns |
| Control at 2 p.m., HDT, HNDT | Genotype | 14.77*** | 2.88*** | 12.93*** | 2.81*** |
| | Treatment | 11.44*** | 2.23*** | 10.02* | 2.18*** |
| | Stage | 11.44*** | 2.23*** | 10.02*** | 2.18*** |
| | Genotype × Treatment | ns | 4.99*** | ns | 4.87* |
| | Genotype × Stage | 25.58*** | 4.99*** | 22.40*** | 4.87*** |
| | Treatment ×Stage | 19.81*** | 3.87*** | ns | 3.77*** |
| | Genotype × Treatment × Stage | 44.30*** | 8.65*** | ns | 8.43*** |

Supplementary Table S5. Correlation coefficients of the activities of four enzymes taken at 4 a.m. on 5, 10, 15 days after flowering (DAF), including cell wall invertase, vacuolar invertase, sucrose synthase, or starch synthase, with final grain weight, non-structural carbohydrates (NSC) in the grains, and grain-filling parameter values derived through the sigmoid grain growth equation ($C_m$, the maximum grain filling rate; $\bar{C}$, mean grain filling rate; $t_m$, time when the maximum growth rate is achieved; $t_e$, the time at which the maximum of grain weight is reached.), in five rice genotypes exposed to control (31 °C/23 °C (day/night)) and higher night-time temperature (HNT-31 °C/30 °C) at grain filling stage lasting for 20 days.

| Days after flowering (DAF) | Enzyme | Grain weight | NSC | $C_m$ | $\bar{C}$ | $t_m$ | $t_e$ |
|---|---|---|---|---|---|---|---|
| 5 DAF | Cell wall invertase | -0.230 | -0.718* | 0.019 | -0.189 | 0.622* | -0.118 |
| | Vacuolar invertase | -0.107 | -0.755** | -0.184 | -0.340 | 0.682* | 0.060 |
| | Sucrose synthase | 0.637* | -0.391 | -0.852*** | -0.759** | 0.329 | 0.896*** |
| | Starch synthase | -0.078 | -0.767** | -0.200 | -0.348 | 0.701* | 0.088 |
| 10 DAF | Cell wall invertase | -0.554 | 0.082 | 0.655* | 0.459 | -0.047 | -0.663* |
| | Vacuolar invertase | -0.432 | 0.059 | 0.666* | 0.557 | 0.081 | -0.663* |
| | Sucrose synthase | -0.273 | 0.006 | 0.141 | 0.244 | -0.287 | -0.230 |
| | Starch synthase | -0.464 | 0.020 | 0.660* | 0.522 | 0.104 | -0.666* |
| 15 DAF | Cell wall invertase | 0.091 | -0.263 | -0.240 | -0.371 | 0.422 | 0.295 |
| | Vacuolar invertase | -0.070 | -0.450 | -0.330 | -0.490 | 0.708* | 0.181 |
| | Sucrose synthase | -0.376 | -0.162 | 0.458 | 0.474 | -0.881** | -0.459 |
| | Starch synthase | -0.079 | -0.496 | -0.410 | -0.560 | 0.705* | 0.230 |

*,**,*** significant at the 0.05, 0.01 and 0.001 probability level, respectively. df=9

Supplementary Table S6. Correlation coefficients of the activities of four enzymes taken at 2 p.m. on 5, 10, 15 days after flowering (DAF), including cell wall invertase, vacuolar invertase, sucrose synthase, or starch synthase, with final grain weight, non-structural carbohydrates (NSC) in the grains, and grain-filling parameter values derived through the sigmoid grain growth equation ($C_m$, the maximum grain filling rate; $\bar{C}$, mean grain filling rate; $t_m$, time when the maximum growth rate is achieved; $t_e$, the time at which the maximum of grain weight is reached), in five rice genotypes exposed to control (31 °C/23 °C (day/night)), higher day-time temperature (HDT-38 °C/23 °C) or combined higher night-time and day-time temperature (HNDT-38 °C/30 °C) at grain filling stage lasting for 20 days.

| Days after flowering (DAF) | Enzyme | Grain weight | NSC | $C_m$ | $\bar{C}$ | $t_m$ | $t_e$ |
|---|---|---|---|---|---|---|---|
| 5 DAF | Cell wall invertase | 0.245 | -0.372 | 0.255 | 0.290 | 0.152 | 0.000 |
| | Vacuolar invertase | 0.402 | -0.412 | 0.186 | 0.256 | 0.288 | 0.149 |
| | Sucrose synthase | -0.494 | -0.001 | -0.545* | -0.597* | -0.287 | -0.089 |
| | Starch synthase | 0.396 | -0.426 | 0.172 | 0.243 | 0.296 | 0.153 |
| 10 DAF | Cell wall invertase | -0.381 | 0.390 | 0.667** | 0.586* | -0.552* | -0.701** |
| | Vacuolar invertase | -0.205 | 0.466 | 0.676** | 0.636** | -0.460 | -0.568* |
| | Sucrose synthase | -0.195 | 0.247 | 0.283 | 0.239 | -0.332 | -0.292 |
| | Starch synthase | -0.419 | 0.467 | 0.624** | 0.607* | -0.698** | -0.713** |
| 15 DAF | Cell wall invertase | 0.695** | 0.550* | -0.111 | 0.033 | 0.371 | 0.628** |
| | Vacuolar invertase | 0.393 | 0.354 | 0.162 | 0.267 | -0.062 | 0.257 |
| | Sucrose synthase | -0.519* | -0.396 | 0.487 | 0.400 | -0.664** | -0.656** |
| | Starch synthase | 0.543* | 0.384 | 0.003 | 0.095 | 0.185 | 0.462 |

*,** significant at the 0.05 and 0.01 probability level, respectively. df=14

Supplementary Figure S1. Scanning electron microscopic observation of the transverse section of the central part of the developing grains collected at 5 days after flowering in four rice genotypes exposed to control (31 °C/23 °C (day/night)), higher night-time temperature (HNT-31 °C/30 °C), higher day-time temperature (HDT-38 °C/23 °C) or combined higher night-time and day-time temperature (HNDT-38 °C/30 °C) at grain filling stage for 20 days after flowering. Magnification = ×2,000. Yellow arrows indicate the single granules. Red arrows indicate the single granules grouping into amyloplast.

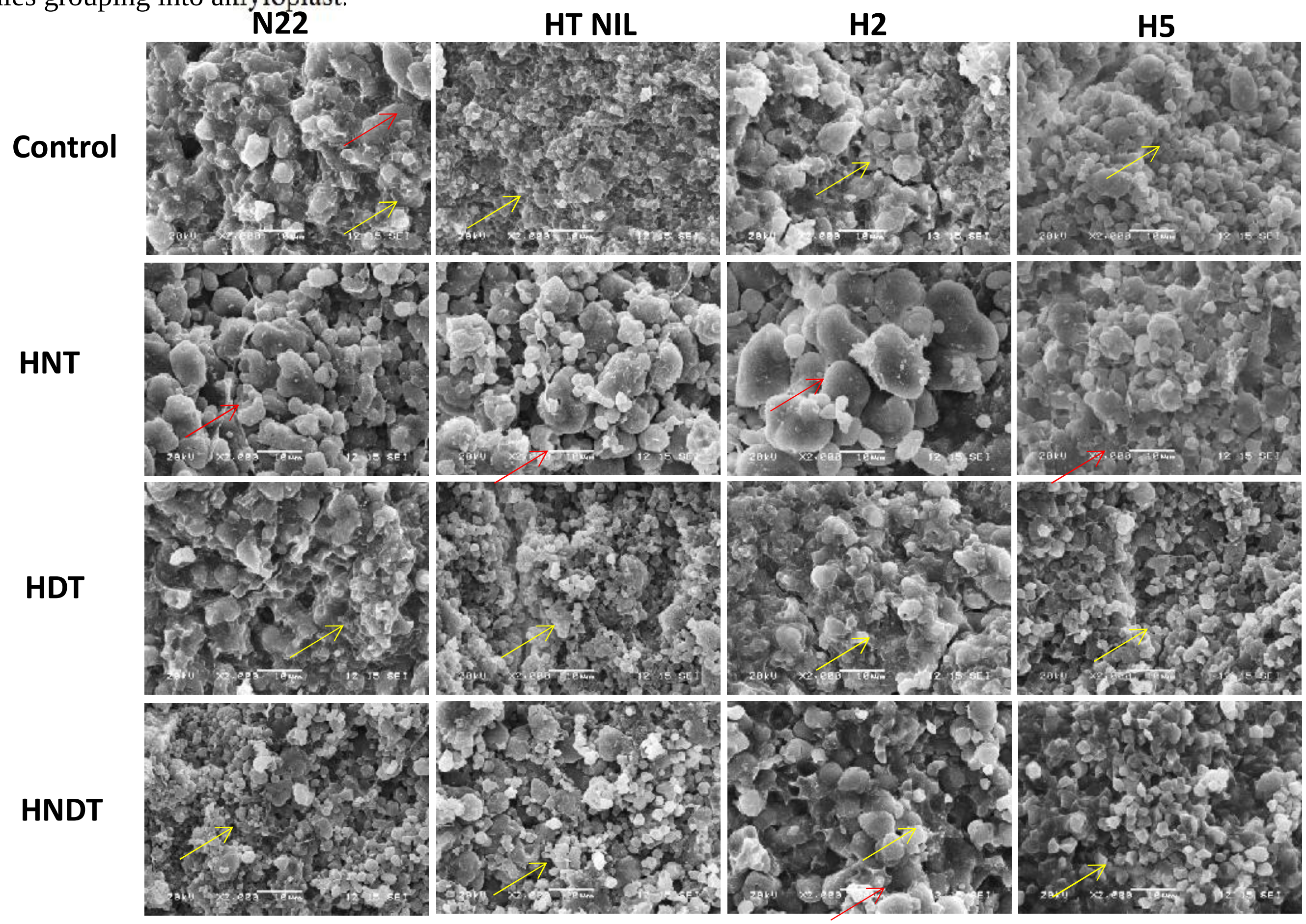

Supplementary Figure S2. Scanning electron microscopic observation of the transverse section of the central part of the developing grains collected at 10 days after flowering in four rice genotypes exposed to control (31 °C/23 °C (day/night)), higher night-time temperature (HNT-31 °C/30 °C), higher day-time temperature (HDT-38 °C/23 °C) or combined higher night-time and day-time temperature (HNDT-38 °C/30 °C) at grain filling stage for 20 days after flowering. Magnification = ×2,000. Red rectangle indicates the polygonal shape of starch granules grouping into amyloplast without airspaces. Yellow elliptical ring shows poorly developed amyloplasts together with the individual round shape and heterogeneous size of starch granules large airspaces between.

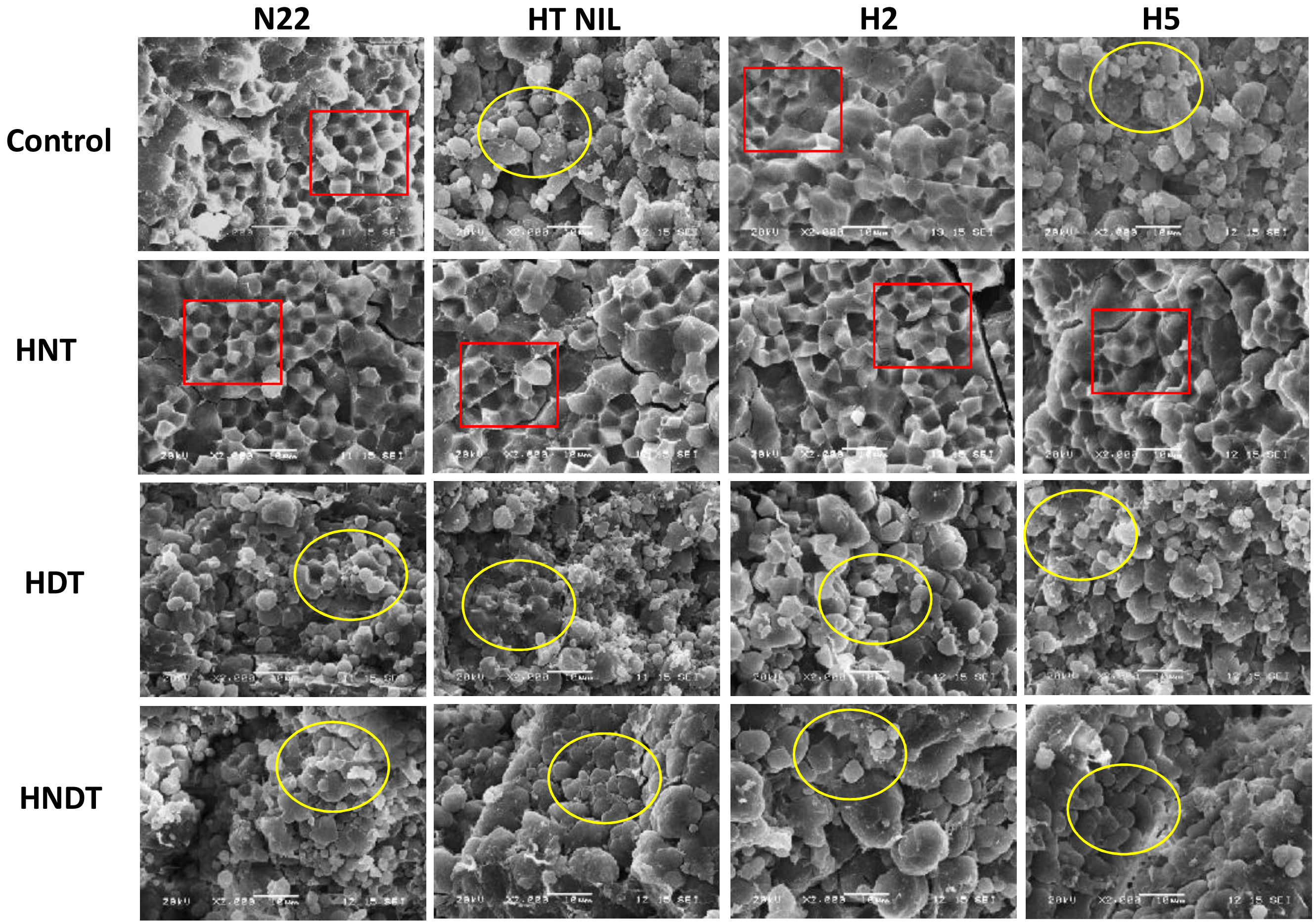

Supplementary Figure S3. Scanning electron microscopic observation of the transverse section of the central part of the developing grains collected at 15 days after flowering in four rice genotypes exposed to control (31 °C/23 °C (day/night)), higher night-time temperature (HNT-31 °C/30 °C), higher day-time temperature (HDT-38 °C/23 °C) or combined higher night-time and day-time temperature (HNDT-38 °C/30 °C) at grain filling stage for 20 days after flowering. Magnification = ×2,000. Red rectangle indicates the polygonal shape of starch granules grouping into amyloplast without airspaces. Yellow elliptical ring shows poorly developed amyloplasts together with the individual round shape and heterogeneous size of starch granules large airspaces between.

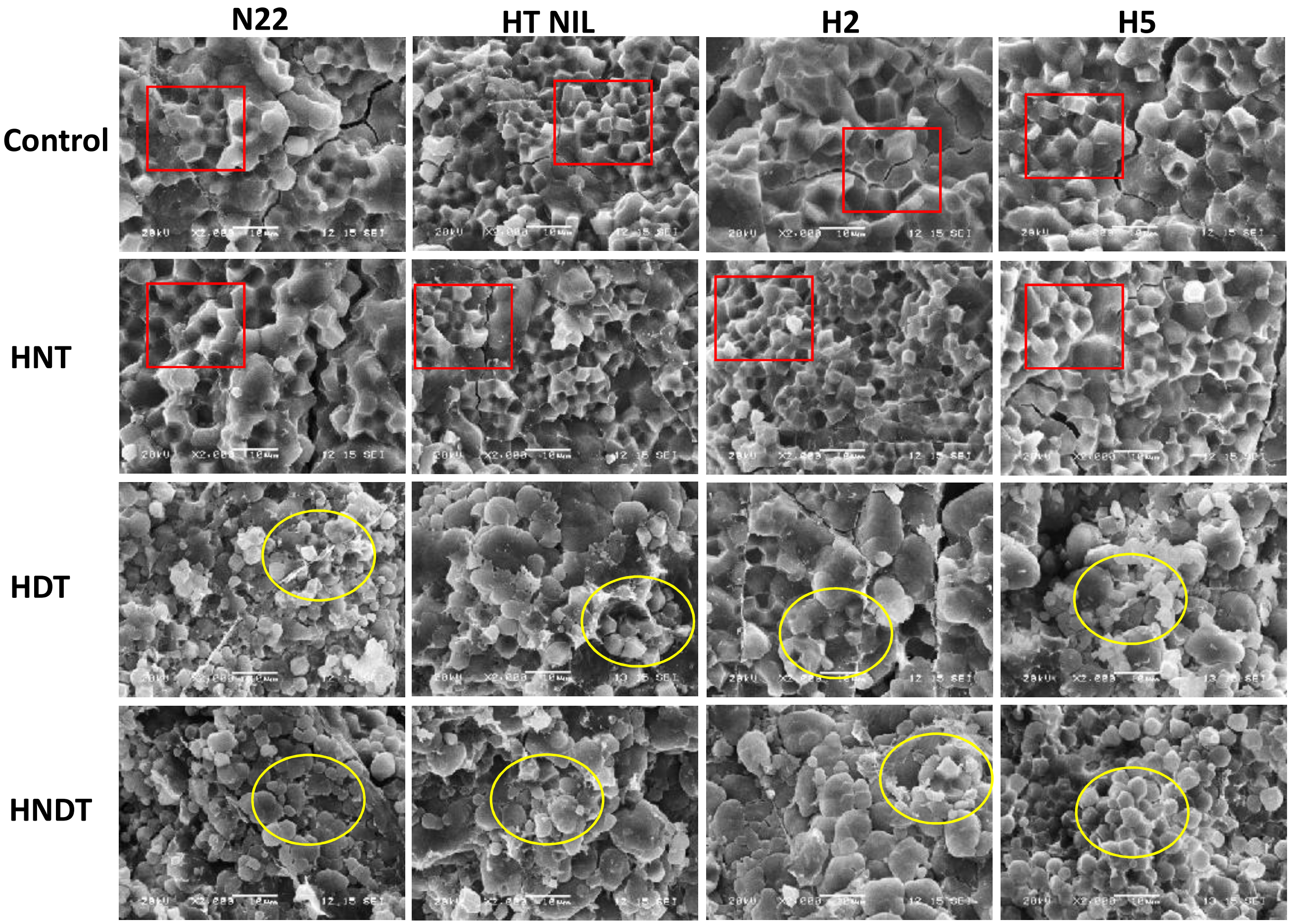

Supplement: supplementary_tables_S1_S6_Figures_S1_S3 [file erx344_suppl_supplementary_tables_s1_s6_figures_s1_s3.pdf]
